# Influence of Acute Normobaric Hypoxia on Hemostasis in Volunteers with and without Acute Mountain Sickness

**DOI:** 10.1155/2015/593938

**Published:** 2015-09-15

**Authors:** Marc Schaber, Veronika Leichtfried, Dietmar Fries, Maria Wille, Hannes Gatterer, Martin Faulhaber, Philipp Würtinger, Wolfgang Schobersberger

**Affiliations:** ^1^Institute for Sports Medicine, Alpine Medicine and Health Tourism, University for Health Sciences, Medical Informatics and Technology (UMIT), Eduard-Wallnöfer-Zentrum 1, 6060 Hall, Austria; ^2^Department for General and Surgical Critical Care Medicine, Medical University of Innsbruck, Anichstrasse 35, 6020 Innsbruck, Austria; ^3^Department of Sports Science, Leopold Franzens University Innsbruck, Fürstenweg 185, 6020 Innsbruck, Austria; ^4^Central Institute for Medical and Chemical Laboratory Diagnosis, Medical University of Innsbruck, Anichstrasse 35, 6020 Innsbruck, Austria; ^5^Institute for Sports Medicine, Alpine Medicine and Health Tourism, Tirol Kliniken Innsbruck and University for Health Sciences, Medical Informatics and Technology (UMIT), Eduard-Wallnöfer-Zentrum 1, 6060 Hall, Austria

## Abstract

*Introduction*. The aim of the present study was to investigate whether a 12-hour exposure in a normobaric hypoxic chamber would induce changes in the hemostatic system and a procoagulant state in volunteers suffering from acute mountain sickness (AMS) and healthy controls. *Materials and Methods*. 37 healthy participants were passively exposed to 12.6% FiO_2_ (simulated altitude hypoxia of 4,500 m). AMS development was investigated by the Lake Louise Score (LLS). Prothrombin time, activated partial thromboplastin time, fibrinogen, and platelet count were measured and specific methods (i.e., thromboelastometry and a thrombin generation test) were used. *Results*. AMS prevalence was 62.2% (LLS cut off of 3). For the whole group, paired sample *t*-tests showed significant increase in the maximal concentration of generated thrombin. ROTEM measurements revealed a significant shortening of coagulation time and an increase of maximal clot firmness (InTEM test). A significant increase in maximum clot firmness could be shown (FibTEM test). *Conclusions*. All significant changes in coagulation parameters after exposure remained within normal reference ranges. No differences with regard to measured parameters of the hemostatic system between AMS-positive and -negative subjects were observed. Therefore, the hypothesis of the acute activation of coagulation by hypoxia can be rejected.

## 1. Introduction

Acute, high altitude exposure induces a large variety of adaptive mechanisms in the nonadapted human body. Currently, the main research focus has been on the physiology and pathophysiology of the cardiovascular, cerebral, and pulmonary systems, including maladaptation, in acute mountain sickness [1, 2]. There have only been few studies with a special focus on hypoxia-induced changes of plasma coagulation, fibrinolysis, and platelet function.

Most information about acute hypoxia and hemostatic changes has been obtained by studies focusing on long-haul flights and travel thrombosis. However, these data are inconsistent [3–7] and the results cannot be easily transferred to high altitude physiology since hypoxia during aircraft travel is moderate (maximum corresponding to an altitude of 2,500 m) and sitting in a cramped position itself may be a central trigger for coagulation changes.

Even studies focusing on high altitude are nonuniform. Maher et al. [8] investigated several parameters of coagulation and platelet aggregation during simulated high altitude exposure (4,400 m) and found some parameters to be changed indicative for a coagulopathy. O'Brodovich et al. [9] reported hemostatic changes after acute exposure to hypobaric and normobaric hypoxia (inspired fraction of oxygen, FiO_2_ = 0.11), showing shortening of activated partial thromboplastin time (aPTT) and an increase in procoagulant plasma factor VIII:C-like activity. In contrast, Bärtsch et al. [10] could not show any changes in fibrin or thrombin formation during a 22-hour ascent from 3,200 m to 4,559 m. A prothrombotic state was reported by Mannucci et al. [11] in unacclimatized subjects who were transported by helicopter after a 48-hour stay from 1,200 m to 3,940 m and after another 24-hour stay were transported to 5,060 m.

At high and extreme altitudes, subjects are exposed to a variety of factors which could influence the hemostatic system (e.g., cold, dehydration, polyglobulia, immobility during periods of bad weather, and exhaustive physical exercise). Since decades, thrombotic and thromboembolic events have been described in climbers [12–16]. However, all reports were either case reports or retrospective observations; therefore, the prevalence of high altitude associated thromboembolism remains unclear. In addition, several mountaineers suffering from thrombosis had individual risk factors (e.g., oral contraceptives, genetic mutations as factor V Leiden mutation, and prothrombin polymorphism). Therefore, the impact of hypoxia itself as an independent risk factor for thrombosis at high altitude is still a matter of debate [17–19].

Hypoxic chamber studies appear to be an effective and valid method to investigate acute mountain sickness (AMS), since AMS not only manifests in nonacclimatized trekkers and mountaineers who rapidly ascent to altitudes above 2,500 m [20] but is also a frequent health-related problem for subjects in hypoxic chamber studies [21, 22]. Nevertheless, the link between acute hypoxia, AMS formation, and hemostasis is still unknown. Bartsch et al. [23] showed that, after climbing an altitude of 4,559 m, factor VIII procoagulant activity and von Willebrand factor antigen were increased in AMS-positive subjects, whereas Pichler Hefti et al. [24] were unable to detect any association between AMS scores and coagulation parameters.

To evaluate the effects on procoagulants by acute and chronic hypoxia [9, 25], standard laboratory tests like prothrombin time (PT) and aPTT seem to be inferior compared to methods like thromboelastography (TEG) [26] or thrombin generation [27]. In 2012, TEG was used for the first time at high altitude settings (5,300 m). After a 13-day standardized ascent profile (from 2,800 m to 5,300 m), TEG results showed reduced coagulation activation in healthy volunteers [26]. However, studies under standardized acute hypoxic conditions using TEG and thrombin generation are still lacking. Therefore, in the present study, hemostasis was analyzed by applying TEG and thrombin generation during a simulated acute hypoxic setting, in which subjects reached altitudes similar to high altitude tours and where confounding variables like cold, dehydration, and prolonged immobility could be ruled out due to the study design.

We hypothesized that a 12-hour sojourn in a hypoxic chamber corresponding to 4,500 m would provoke the activation of hemostasis in nonacclimatized healthy volunteers. In addition, we speculated that this coagulation activation is more pronounced in volunteers who develop AMS during hypoxia as compared to those who do not.

## 2. Materials and Methods

### 2.1. Participants

The present study was part of a large, simulated, high altitude project performed in Innsbruck, Austria. Parts of the project have already been published [21]. Participants were mainly recruited via advertisements on the homepage of the Austrian Alpine Association and information via the mailing list of the University of Innsbruck. Exclusion criteria were pregnancy, reported cardiovascular, respiratory, neurological, and psychiatric diseases, migraine, chronic headache, smoking, permanent residence at altitudes exceeding 1,000 m, an overnight stay at altitudes greater than 2,500 m in the previous month, or exposure above 2,500 m for 2 weeks prior to the 12-hour hypoxic exposure. Participants were instructed to abstain from all anti-inflammatory medications and nutritional supplements for 2 weeks prior to the exposure and from alcohol starting the day before the experiment. Caffeine was not allowed on the day of the exposure.

All participants gave their written informed consent prior to participation in the study. The study was carried out in conformity with the ethical standards laid down in the 2008 Declaration of Helsinki and was approved by the Ethics Committee of the Medical University of Innsbruck (program code: UN4522, session: 306/4.11).

### 2.2. Procedures

Participants were passively exposed to a FiO_2_ of 12.6% (corresponding to a simulated altitude hypoxia of 4,500 m at 590 m, PiO_2_ = 83.9 mmHg) for 12 hours. Room temperature and humidity were kept constant at 22–24°C and 23–27%, respectively. Prior to entering the hypoxic chamber, participants were examined, including a medical routine check. During the stay in the chamber, food (e.g., brown bread, cheese, boiled ham, cucumber, banana, apple, cookies, and chocolate) and drinks (water and apple juice) were provided ad libitum. Most of the time, participants stayed seated, but some activities (e.g., standing, walking, and stretching) were also performed. Recumbent position or sleeping was not allowed.

### 2.3. Measurements and Instruments

#### 2.3.1. Lake Louise Score (LLS) and AMS

To assess the prevalence and severity of AMS [28], the LLS was used. It is a self-assessment questionnaire including five symptom complexes (headache; gastrointestinal symptoms like anorexia, nausea, or vomiting; fatigue and/or weakness; dizziness and/or light headedness; and difficulty of sleeping); scores range from 0 to 3. The subjects self-rated their status: 0 for no discomfort and 1 for mild, 2 for moderate, and 3 for severe symptoms. Since participants did not stay overnight in the hypoxic chamber, the symptom complex “difficulty sleeping” was not taken into account. AMS was diagnosed when the symptom headache and at least one other symptom were present, with a total score of at least 3. Scores did not distinguish between mild and severe forms of AMS [29]. The LLS was assessed before entering the chamber and after 3, 6, 9, and 12 hours in the chamber or when participants left the chamber at an earlier time point. The maximum AMS score was used to distinguish between AMS-positive (AMS+) and AMS-negative (AMS−) volunteers.

Arterial oxygen saturation (SpO_2_) and heart rate were measured using pulse oximetry (Onyx II 9550, NONIN, Plymouth, MI, USA) after 0.5, 3, 6, 9, and 12 hours in the chamber.

#### 2.3.2. Hematological Parameters, Thromboelastometry (ROTEM), and Thrombin Generation

Venous blood samples were taken immediately before and at the end of the 12-hour hypoxic exposure or when participants left the chamber at an earlier time point. Blood processing was done immediately thereafter in the hemostasis laboratory.

Blood was drawn in ethylenediaminetetraacetic acid-containing tubes (Sarstedt, Nümbrecht, Germany) for platelet counts and in sodium citrate-containing tubes (Sarstedt) for coagulation assays. Platelet counts were determined using the Sysmex XE 5000 hematology analyzer (Sysmex Corporation, Kobe, Japan).

Platelet-poor plasma for the determination of plasma coagulation tests was obtained by centrifugation at 2,100 ×g for 15 min and another centrifugation at 10,000 ×g for 5 min.

Parameters of aPTT, PT, and Clauss fibrinogen assay results [30] were determined on a BCS-XP instrument (Siemens Healthcare Diagnostics, Marburg, Germany) and an automated coagulation analyzer.

In ROTEM, the resistance of a rotated pin in a stationary cuvette, filled with citrated whole blood, is measured after coagulation activation with different reagents. For additional monitoring of routine coagulation tests in subjects, the following parameters were automatically detected by the ROTEM analysis software based on thromboelastograms: clotting time (CT), clot formation time (CFT), alpha angle, maximum clot firmness (MCF) after 15 min in ExTEM (activation of coagulation via tissue factor) and INTEM (activation of coagulation via the contact phase), and MCF after 15 min in FIBTEM (activation of coagulation via tissue factor and cytochalasin D for the inhibition of platelets). Although the ROTEM system was mainly established and used for the control and differential diagnosis of hemostatic disorders within the context of acute bleeding, recent literature has also suggested a possible role for the ROTEM system in testing for hypercoagulable states [5, 24]. Additional information about ROTEM techniques, its parameters, and principles has been described in detail elsewhere [31–34].

Thrombin generation analysis was performed with platelet poor plasma using the Innovance ETP assay (Siemens) on a BCS XP automated coagulation analyzer (Siemens). After the activation of coagulation with synthetic phospholipids [35], human recombinant tissue factor (Innovin; BC Siemens), and calcium ions in the absence of thrombomodulin, a chromogenic substrate H-b-Ala-Gly-Arg-pNA is cleaved by the generated thrombin. The final concentration of substrate is 733 nmol/L and 19 mmol/L CaCl. The concentration of phospholipids and tissue factor is confidential to the manufacturer. To prevent fibrin polymerization, the reagent contains an undefined clot inhibitor. The conversion of the slow-reacting chromogenic substrate is detected over time at a wavelength of 405 nm. After correction for the *α*-macroglobulin-bound thrombin activity, the thrombin generation curve can be obtained. From this curve, the total amount of generated thrombin referred to as “endogenous thrombin potential” (area under the curve, ETP AUC), the peak thrombin generation (*C*_max⁡_), the lag phase until initiation (*t*_lag_), and the time to peak thrombin activity (*t*_max⁡_) can be determined. The concentration of generated thrombin (*C*_max⁡_) (mE/min) is the maximum of the first derivation of the ETP AUC. It corresponds with the maximal thrombin generation. These two values were calibrated against a normal human plasma pool (Innovance ETP Standard, BC Siemens) and then expressed as a percentage. The time needed until the first thrombin activity is registered is the lag time (*t*_lag_) (sec). The time to reach the maximum peak, which constitutes the maximum thrombin generation, is called *t*_max⁡_ (sec) [36].

### 2.4. Statistical Analysis

All participants who endured the simulated exposure to high altitude for 12 hours without suffering severe symptoms were included in the statistical analysis. Others were counted as dropouts. Box plots were used to determine whether data distributions were symmetrical. Parametric tests were used for normally distributed data, and nonparametric tests were used for skew data. Results from categorical variables are reported as proportions, and continuous variables are reported as means ± standard deviation (SD). Comparisons of parameters before and after simulated altitude exposure were made using the Student's *t*-test (normally distributed data) and Wilcoxon test (skew data) for matched pairs. Comparison of change scores (before hypoxia and after hypoxia) between participants suffering from AMS (LLS ≥ 3; AMS+) and those not suffering from AMS (LLS < 3; AMS−) was made using the Student's *t*-test (normally distributed data) and Mann-Whitney *U* test (skew data) for unmatched pairs. Spearman's rank correlation coefficients (*r*_S_) of all blood parameters were evaluated.

Changes over time in heart rate and SpO_2_ values, as well as location of changes over time, were calculated using linear models for repeated measurements. Significant values were adapted using Bonferroni's correction. Two-tailed *P* values less than 0.05 were determined to be significant for all statistical evaluations.

All statistical calculations were made using IBM SPSS Statistics, version 20.0 (Chicago, IL, USA).

## 3. Results

No serious or unexpected adverse events were observed during the chamber stay.

### 3.1. Anthropometric Data and Baseline Characteristics

In total, 37 participants were included in the statistical analysis (Table 1). Sixteen females and twenty-one males participated. The average age of all participants was 25.9 ± 5.6 years (range, 19 to 42 years). Body height varied from 160 cm to 197 cm (mean, 174 ± 9 cm) and body weight from 42.8 kg to 88.3 kg (mean, 67 ± 11 kg). Body mass index (BMI) was 22.0 ± 2.3 (range, 15.7 to 26.5). The amount of exercise performance per week was 8.1 ± 4.7 hours (range, 1–25 hours). Of the female participants, 33.3% (*n* = 5) took oral contraceptives.

Both groups (AMS−/AMS+) were homogeneous, and no significant differences were shown for age (*P* = 0.690), body height (*P* = 0.072), or body weight (*P* = 0.375). AMS+ group was comprised of eleven male and twelve female participants while the AMS− group included ten male and four female subjects.

### 3.2. AMS Prevalence

One participant who discontinued chamber exposure after 5 hours and 40 min was classified as a dropout, because he showed no symptoms of AMS and terminated for unknown reasons. Five subjects were excluded as dropouts as a result of preanalytical errors such as insufficient blood sample volumes or missing AMS data. Due to severe symptoms, nine participants (three male and six female subjects) left the chamber before the end of the 12 hours (mean LLS = 5.2 ± 1.4). The analysis of all included volunteers (*n* = 37) resulted in an AMS prevalence of 62.2% (*n* = 23, on basis of the overall maximum AMS score of ≥ 3); 14 subjects (37.8%) did not show any signs of AMS (in total minimum LLS = 0, maximum LLS = 8, and mean LLS = 3.4 ± 2.1; *n* = 37). Of the 28 subjects who completed the full 12-hour session in the chamber, 19 subjects (67.9%) were AMS− and 9 (32.1%) were AMS+ (minimum LLS = 0, maximum LLS = 6, and mean LLS = 1.79 ± 1.89; *n* = 28).

### 3.3. Peripheral Capillary Oxygen Saturation (SpO_2_) and Heart Rate (HR)

Whole-group data showed that both HR and SpO_2_ significantly changed over time (HR from 76 ± 12 bpm to 84 ± 15 bpm, *P* = 0.008, and SpO_2_ from 98.1 ± 1.2% to 85.1 ± 6.2%, *P* < 0.000). For SpO_2_ values, a significant decrease occurred in the first one-half hour of hypoxic exposure (*P* < 0.000) but showed no further decrease afterwards.

HR was significantly higher after 3 hours of hypoxic exposure compared to preexposure values (*P* = 0.026) and stayed constant thereafter. There was no difference in HR_max⁡_ values during hypoxic exposure between AMS+ and AMS− subjects (*P* = 0.251). Comparison of SpO_2_  (SpO_2max_ − SpO_2min_) during the chamber exposure between AMS+ and AMS− subjects revealed no differences (*P* = 0.801).

### 3.4. Standard Parameters of Hemostasis, Thrombin Generation, and Thromboelastometry

#### 3.4.1. aPTT and PT

All baseline data of the aPTT and PT analysis were within the reference range. There were no changes in aPTT for the whole group or for AMS+ and AMS− subjects during hypoxic exposure. PT was increased after the chamber sojourn in the AMS− group only. A comparison of the differences (data before and after the chamber session) of the two populations resulted in a significant ΔPT (*P* = 0.035; AMS−  6.1 ± 9.3%; AMS+ 0.4 ± 6.4%).

#### 3.4.2. Platelet Count and Fibrinogen

Pooled data did not show any changes in platelet counts or fibrinogen, and baseline data stayed within reference ranges (Table 2). In subjects with AMS, no differences in pre- or posthypoxic exposure values could be shown for either parameter. In contrast, a significant increase in platelet count was detected in participants without AMS after chamber exposure.

#### 3.4.3. Thrombin Generation

All baseline data from the thrombin generation analysis were within the reference range (Table 3). With the exception of *C*_max⁡_, where an increase in the whole group was observed, no significant changes were measured for any other thrombin generation parameters independent of subgroup before or after hypoxia.

#### 3.4.4. ROTEM Measurements

ROTEM baseline data measurements changed within reference ranges (Table 4). When comparing baseline values of the AMS+ and AMS− groups, only the ROTEM parameters of MCF InTEM (*P* = 0.023) and MCF ExTEM (*P* = 0.029) showed significant differences between pre- and postexposure. After hypoxia in the InTEM analysis, significant shortening of CT (ΔCT = −6.3 ± 15.5 sec) and an increase in MCF (ΔMCF = 0.95 ± 2.7 mm) were found for the whole group. Furthermore, a significant increase in MCF (ΔMCF = 0.87 ± 2.4 mm) could be shown in the FibTEM test. A comparison of the differences between the means (data before and after the chamber session) of the two populations showed significant differences for ΔMCF InTEM (*P* = 0.023; AMS−  2.21 ± 2.5 mm; AMS+ 0.17 ± 2.6 mm) and ΔMCF ExTEM (*P* = 0.029; AMS−  1.36 ± 3.4 mm; AMS+ −1.22 ± 3.3 mm). A tendency for a change in CFT (*P* = 0.057; AMS−−7.79 ± 16.6 sec; AMS+ 1.52 ± 12.1 sec) in the InTEM analysis was observed.

The CT InTEM analysis showed a significant shortening in AMS+ subjects (ΔCT = −7.1 ± 12.2 sec), and MCF increased in AMS− subjects in both the InTEM (ΔMCF = 2.2 ± 2.5 mm) and FibTEM analyses (ΔMCF = 1.3 ± 1.5 mm).

### 3.5. Correlations

When assessing the relationship between LLS max and the laboratory parameters, a Spearman correlation coefficient of 0.329 (*P* = 0.046) between LLS max and ΔPT was found. A negative Spearman correlation coefficient was measured for ΔCFT InTEM and LLS max (−0.447; *P* = 0.006) and for ΔMCF InTEM and LLS max (−0.413; *P* = 0.011). Furthermore, a correlation between ΔMCF ExTEM and LLS max was found (0.399; *P* = 0.014).

## 4. Discussion

The aim of the study at hand was to investigate possible hypoxia-induced changes in hemostasis in primary healthy volunteers during short-term (12 hours) exposure in a normobaric hypoxic chamber. Furthermore, it was hypothesized that subjects developing AMS would exhibit an activation of coagulation. However, during the 12-hour hypoxic exposure, only a few, small changes in the routine as well as in the specialized parameters of coagulation and fibrinolysis could be detected. Moreover, no significant differences in key parameters between volunteers who developed AMS and those who did not were measured.

The present chamber study simulated an altitude of 4,500 m, which was sufficient to provoke AMS even within 12 hours. The overall prevalence of AMS was 62.2%, which proved that our setting was adequate not only to investigate possible coagulation alterations for the whole group of participants but also to detect group differences (AMS+ versus AMS−). There are different approaches to investigate AMS and its consequences in controlled settings. Commonly used methods are chamber decompression to generate hypobaric hypoxia or adjustments for oxygen levels for normobaric hypoxia [37, 38]. MacInnies et al. [37] exposed 25 subjects to a partial pressure of inspired oxygen of 90 mmHg (4,000 m equivalent) and found AMS prevalence of 84% and 56% during two separate, 12-hour night sessions, which are similar to our results and those of other studies performed in high-altitude environments [39, 40].

Our unacclimatized participants showed reduced peripheral capillary SpO_2_ and increased HR in the chamber. These results are in accordance with others who reported an activation of the sympathetic nervous system during acute exposure to high altitude, which was evident not only during environmental exposure but also in hypobaric chambers [41–43]. Faulhaber et al. [22] were able to demonstrate that SpO_2_ measurements after 30 min of hypoxic exposure have the potential to detect AMS-susceptible individuals. Karinen et al. [44] also showed that reduced SpO_2_ during resting and after exercise measured at altitudes of 3,500 m and 4,300 m seems to predict AMS at high altitudes. In a recent meta-analysis of 12 studies, a significant association between differences in SpO_2_ and the risk of developing AMS was reported [45]. However, SpO_2_ and HR did not differ during hypoxia between AMS+ and AMS− subjects; thus, SpO_2_ had no predictive value at least in our setting.

Currently, the majority of publications related to hemostatic alterations in hypoxia have not been based on high altitude but on travel medicine, that is, travel-related thromboembolism. Studies were performed either under simulated moderate hypoxic conditions or during situations of long-distance travel (flights or bus travel). The corresponding data are conflicting and results vary from unchanged coagulation [6, 7, 46] to activation of coagulation and/or suppression of fibrinolysis [3, 4, 47] in healthy subjects. A few studies even reported a reduced thrombin generation in hypoxia [48, 49]. Although a few aspects of long-haul flights might be applicable to our study, the scientific approach is different. The degree of hypoxia is reduced during long-haul flights and in other chamber studies focusing on hemostasis compared to our study. In all travel-related studies, the participants were seated in a more-or-less cramped position that may worsen leg venous blood flow, thus triggering coagulation activation [50]. In our chamber study, the volunteers were able to sit comfortably and allowed to move freely during the chamber sojourn. Therefore, blood stasis in the lower legs can be excluded.

Data on hemostasis during real ambient hypoxia at high altitudes is scarce and more or less inconsistent. Only a few studies that focused on coagulation changes within a few hours of hypoxia are available. After a 22-hour ascent to 4,559 m Bärtsch et al. reported only a slight increase in PF1+2 with no evidence of significant thrombin or fibrin formation [10]. Similar results were obtained for TAT, PF1+2, and fibrinopeptide A in healthy mountaineers after a 2-3-day walk to high altitudes [23]. In contrast, a prothrombotic state (increase in PF1+2 and PAI-1 activity and antigen) was reported in unacclimatized mountaineers after passive transport by helicopter from 1,200 m to 5,060 m within 2 days [11].

By pooling all data of the participants, independent of developing AMS, only a few significant changes in the measured hemostatic parameters were detected. In detail, in the thrombin generation analysis *C*_max⁡_ was increased, the ROTEM CT InTEM was shortened, and MCF InTEM and MCF FibTEM were both increased. Recently, peak thrombin generation and ETP AUC were used as predictors of venous thromboembolism [51, 52]. Additionally, in a cell-based model of coagulation, the influence of coagulation factors, which are involved in the formation of the tenase complex (factor (F)VIII, factor (F)IX, and factor (F)XI), on *C*_max⁡_ was demonstrated [53]. Therefore, thrombin generation is thought to be an appropriate tool for the detection of hypercoagulation. Although the ROTEM system is currently established and used for the control and differential diagnosis of hemostatic disorders within the context of acute bleeding, recent literature has also suggested a possible role for the ROTEM system in testing for hypercoagulable states [33, 34]. However, the absolute changes in the present study were small, and all parameters remained within the reference limits. The ROTEM results at hand are not consistent with those of Martin et al. [26], who reported reduced coagulation at high altitude identified by increased TEG reaction R-time (similar to CT) and kinetic K-time (similar to CFT). TEG and ROTEM are related tests where the only difference is in the use of the activator, but they do not have completely interchangeable results [54]. However, another reason for the incomparability of the data could be found in the differences of study design: in contrast to our study, the TEG analysis was performed after an ascent profile lasting for several days. Therefore, when interpreting pooled data, present results indicate that hypoxia does not trigger thrombin formation since the total amount of created free thrombin, as measured by ETP AUC which is indicative of maximal thrombin generation, remained unchanged. This finding was supported by the only minor changes in the thromboelastographic parameters.

In order to detect possible effects of hypoxia on AMS genesis, subgroup analyses were performed. In the AMS+ group, no changes in the standard coagulation tests aPTT, PT, platelet count, or fibrinogen were detected. In the AMS− group, PT was shortened and platelet count was increased after hypoxia. ETP AUC remained unchanged in both groups during chamber exposure. Subgroup results of the ROTEM parameters showed shortening of CT InTEM in the AMS+ population and an increase of MCF in the InTEM and FibTEM analysis. Comparing the absolute changes between both groups, no stringent evidence for significant or relevant differences between AMS+ versus AMS− exists.

In case of a strong association between changes in hemostasis and the development of AMS, significant correlations of the maximum LLS during hypoxia with the measured coagulation parameters should have been obtained. However, only a few of the evaluated parameters showed a significant association with maximum LLS (PT, aPTT, CFT InTEM, MCF InTEM, and MCF ExTEM), and correlation coefficients were moderate to low (<0.45) in all cases. This result might indicate the lack of a pathophysiological and clinical relationship between the development of AMS and hypercoagulation.

Limitations of the present study include the relatively small number of participants (*N* = 37), short hypoxic exposure of 12 hours, and the impossibility to include sleep for calculating the original LLS. In addition, the volume of blood collection was limited for coagulation measurements, since it was also used for the determination of other laboratory parameters. This made the analysis of additional parameters for coagulation and fibrinolysis impossible. A further limitation may be the fact that our volunteers were passively exposed to hypoxia. They were allowed to move freely in the chamber, but no additional physical exercise was performed. Therefore, these data cannot be transferred to mountaineering, where both, hypoxia and physical exercise, are usually inseparable. Although it is well known that physical exercise has multiple effects on the hemostatic system, depending on type and intensity of exercise [55], there is no clear evidence that hypoxia per se exacerbates these acute exercise-dependent changes in hemostasis. For example, no differences between normoxic and hypoxic exercises (graded bicycle ergometry) were found for platelet-derived procoagulant microparticles, PDMP-mediated dynamic thrombin generation, and plasma coagulant factors TF, FV, and FVIII [56]. Even a suppression of normoxic exercise-induced increase of procoagulant factors during exercise in hypoxia was published [57].

## 5. Conclusion

In short, the hypothesis of a procoagulant effect of acute hypoxia in healthy individuals was not supported by the present study, since all data remained within normal reference ranges. Furthermore, a clinically relevant alteration of hemostasis in subjects suffering from AMS was not detected during exposure to hypoxia. Therefore, the authors conclude that there is no association in the development of AMS and hypercoagulability.

With respect to high altitude medicine, more studies need to be performed applying new hemostaseological methods that indicate in vivo thrombin formation during longer lasting high altitude exposure.

## Figures and Tables

**Table 1:** Anthropometric data and baseline characteristics.

	All (*n* = 37)	Male (*n* = 21)	Female (*n* = 16)	AMS− (*n* = 14)	AMS+ (*n* = 23)
Age [y]	25.9 ± 5.6	25.3 ± 5.2	26.7 ± 6.1	26.4 ± 3.9	25.6 ± 6.5
Body height [cm]	174 ± 9	179 ± 8	168 ± 5	178 ± 10	172 ± 7
Body weight [kg]	67 ± 11	73 ± 9	60 ± 8	69.7 ± 13.2	66.0 ± 9.3
BMI [kg/m^2^]	22.0 ± 2.3	22.7 ± 2.2	21.2 ± 2.2	21.8 ± 2.8	22.2 ± 1.9

Baseline characteristics of included participants after classification of those without AMS (AMS−) and those suffering from AMS symptoms (AMS+). Data are shown as mean values ± standard deviation.

**Table 2:** Levels of aPTT, PT, platelet count, fibrinogen, SpO_2_, and heart rate.

	ALL				AMS+				AMS−			
	*n*	Before	After	*P*	*n*	Before	After	*P*	*n*	Before	After	*P*
aPTT (26–37 sec)	37	31.5 ± 3.7	30.8 ± 3.4	0.091	23	30.9 ± 3.5	30.1 ± 3.0	0.100	14	32.5 ± 3.9	31.9 ± 3.7	0.489
PT (70–130%)	37	91.7 ± 10.3	94.3 ± 11.2	0.058	23	93.3 ± 8.8	93.7 ± 8.5	0.748	14	89.1 ± 12.3	95.2 ± 15.1^*∗*^	0.029
Platelet count (150–380 G/L)	37	226.0 ± 43.6	232.3 ± 61.4	0.340	23	233.5 ± 36.7	232.3 ± 64.1	0.908	14	213.8 ± 52.2	232.4 ± 58.9^*∗*^	0.000
Fibrinogen (210–400 mg/dL)	37	233.1 ± 36.9	238.6 ± 41.0	0.169	23	238.5 ± 38.8	246.2 ± 45.3	0.104	14	224.1 ± 33.0	226.2 ± 30.3	0.787
SpO_2_ (92–98%)	37	98.1 ± 1.2	85.1 ± 6.2	0.000^*∗*^	23	98.4 ± 1.0	85.3 ± 6.9	0.000	14	97.5 ± 1.5	84.1 ± 6.2	0.000
Heart rate (72–77 bpm)	37	76 ± 12	84 ± 15	0.008^*∗*^	23	79.8 ± 14.3	86.3 ± 16.4	0.011	14	72.6 ± 7.0	81.1 ± 12.0	0.042

aPTT, PT, platelet count, and plasma fibrinogen concentration for all participants (ALL) and for volunteers with (AMS+) and without (AMS−) AMS before and after hypoxia. Data are shown as mean values ± standard deviation.

^*∗*^for *P* < 0.05 as compared to pre exposure. Reference values are given in parentheses.

**Table 3:** Thrombin generation.

	ALL				AMS+				AMS−			
	*n*	Before	After	*P*	*n*	Before	After	*P*	*n*	Before	After	*P*
ETP AUC (76–107%)	37	89.2 ± 10.3	89.9 ± 11.4	0.430	23	90.1 ± 11.0	90.8 ± 11.5	0.474	14	87.9 ± 9.4	88.3 ± 11.5	0.734
*C*_max⁡_ (79–110%)	37	94.0 ± 11.9	100.2 ± 16.7	0.019^*∗*^	23	95.4 ± 12.4	101.7 ± 17.1	0.083	14	91.7 ± 11.0	97.8 ± 16.5	0.122
*t*_lag_ (19.6–25.6 sec)	37	23.9 ± 4.9	23.3 ± 3.8	0.540	23	24.2 ± 5.9	23.8 ± 4.4	0.802	14	23.4 ± 2.4	22.5 ± 2.4	0.143
*t*_max⁡_ (50.8–72.0 sec)	37	66.5 ± 16.9	60.5 ± 7.7	n.a.	23	67.6 ± 18.2	59.7 ± 5.5	n.a.	14	64.7 ± 15.1	61.8 ± 9.8	0.465

n.a.: not applicable.

Endogenous thrombin potential (ETP AUC), maximum concentration of thrombin (*C*_max⁡_), time to peak (*t*_max⁡_), and lag-time (*t*_lag_) for all participants (ALL) and for volunteers with (AMS+) and without (AMS−) AMS before and after hypoxia. Data are shown as mean values ± standard deviation.

^*∗*^for *P* < 0.05 as compared to pre exposure. Reference values are given in parentheses.

**Table 4:** ROTEM measurements.

	ALL				AMS+				AMS−			
	*n*	Before	After	*P*	*n*	Before	After	*P*	*n*	Before	After	*P*
CT InTEM (134–218 sec)	37	156.2 ± 12.8	149.9 ± 16.6^*∗*^	0.012	23	155.7 ± 13.2	148.6 ± 14.4^*∗*^	0.011	14	157.0 ± 12.6	152.1 ± 20.0	0.327
CT ExTEM (42–78 sec)	37	48.0 ± 7.2	49.8 ± 9.8	0.329	23	48.7 ± 7.9	50.2 ± 11.6	0.596	14	46.7 ± 5.9	49.1 ± 6.2	0.283
CFT InTEM (52–116 sec)	37	85.5 ± 17.2	83.5 ± 20.5	0.406	23	80.0 ± 16.1	81.5 ± 18.6	0.552	14	94.6 ± 15.5	86.8 ± 23.7	0.103
CFT ExTEM (53–144 sec)	37	107.6 ± 22.3	107.1 ± 28.4	0.884	23	99.4 ± 18.2	103.6 ± 24.7	0.325	14	121.2 ± 22.3	112.9 ± 33.8	0.237
MCF InTEM (47–69 mm)	37	54.8 ± 4.2	55.8 ± 4.3^*∗*^	0.039	23	55.7 ± 4.4	55.8 ± 4.5	0.749	14	53.5 ± 3.7	55.7 ± 4.1^*∗*^	0.005
MCF ExTEM (48–70 mm)	37	56.9 ± 4.5	56.6 ± 5.1	0.677	23	58.2 ± 4.2	57.0 ± 5.0	0.088	14	54.8 ± 4.2	56.1 ± 5.6	0.163
MCF FibTEM (7–21 mm)	37	11.6 ± 2.4	12.4 ± 2.8^*∗*^	0.035	23	12.2 ± 2.4	12.8 ± 2.9	0.307	14	10.6 ± 2.3	11.9 ± 2.6^*∗*^	0.008

ROTEM analysis before and after chamber exposure for all volunteers (ALL) and according to absence (AMS−) or presence (AMS+) of AMS. ROTEM reference values are given in parentheses. Data are shown as mean values ± standard deviation.

^*∗*^for *P* < 0.05 as compared to pre exposure.
